# Sensorimotor Parameters Predict Performance on the Bead Maze Hand Function Test

**DOI:** 10.3390/s25247670

**Published:** 2025-12-18

**Authors:** Vivian L. Rose, Komal K. Kukkar, Tzuan A. Chen, Pranav J. Parikh

**Affiliations:** 1 Center for Neuromotor and Biomechanics Research, Department of Health and Human Performance, University of Houston, Houston, TX 77204, USA; 2 Department of Psychological, Health, and Learning Sciences, University of Houston, Houston, TX 77204, USA

**Keywords:** force, activity, clinical, hand

## Abstract

Understanding the forces imparted onto an object during manipulation can shed light on the quality of daily manual behaviors. We have developed an objective measure of the quality of hand function in children, the Bead Maze Hand Function Test, which quantifies how well the individual performs the activity by integrating measures of time and force control. Our main objectives were to examine associations between performance (total force output) on the Bead Maze Hand Function Test (BMHFT) and (1) performance on a sensitive measure of force scaling obtained on a laboratory-based dexterous manipulation task, and (2) general sensory and motor parameters important for fine motor skills. A total of 39 typically developing participants ranging in age from 5 to 10 years old (*n* = 28) and 15 to 17 years (*n* = 11). We found that the anticipatory coordination of digit forces was the best predictor of performance on the Bead Maze Hand Function test. We also found that factors such as age, gender, and pinch strength were associated with the BMHFT performance. These findings support the integration of more sensitive sensorimotor metrics, such as the total applied force, into clinical assessments. Linking the development of sensorimotor capabilities to functional task performance may facilitate more targeted and effective intervention strategies, ultimately improving a child’s participation in daily activities.

## 1. Introduction

How a child grasps and manipulates an object depends on the ability to control the forces they use to handle an object [1]. For example, poor control of forces may affect the ability to grade forces while writing, leading to poor handwriting. Similarly, poor force control affects grasping and holding utensils, leading to accidental spills or the inability to complete tasks. Understanding the forces imparted onto an object during manipulation can shed light on the quality of daily manual behaviors [2,3,4,5,6].

Skillful object manipulation depends on predictive (anticipatory) mechanisms formed through experiences as well as reactive mechanisms based on sensory information [1,6,7,8]. Sensory and motor function have been examined during development in terms of their relationships to the control of forces for a grip and lift task [9]. Sensory inputs provide precise information about contact with an object and guide the control of forces while handling an object [3,10]. Distal muscle strength is also important for the application and control of forces [11,12,13,14,15,16]. Typical tests of hand function used in clinics do not examine the control of forces, thus do not objectively assess the sensorimotor behavior, and may be less sensitive to functionally relevant changes in hand function following an intervention [17,18].

Using knowledge from previous laboratory-based studies of force control [19,20,21,22], we have developed an objective measure of the quality of hand function in children, the Bead Maze Hand Function Test (BMHFT) [23]. The BMHFT quantifies not only how quickly a task is accomplished but also how well the individual performs the activity by integrating measures of time and force control. During the test, children use their digits to slide a small bead over smooth wires of different shapes that require small changes in movement direction, while force sensors measure the forces imparted onto the wire. In a previous study, as the wires increased in complexity, participants applied more force as opposed to taking more time when maneuvering the bead [23], indicating that force is a more relevant measure of skill. We suggest that both sensory function and distal strength contribute to the ability to control forces during the BMHFT in children.

The main objective of this study was to examine what sensory and motor parameters best predict the performance (total force output) on the BMHFT in typically developing children. A machine learning-based ridge regression analysis was adopted to allow for the use of correlated variables in the prediction model and prevent overfitting. We considered general sensory and motor parameters important for fine motor skills, such as a sensory stereognosis score, pinch, and key strength, as predictors. We included a sensorimotor integration parameter in the model, measured as the ability to apply (rotational) forces for dexterous manipulation with more mass on one side than the other during a laboratory-based dexterous manipulation task. In the dexterous manipulation task, participants learn to scale the (rotational) forces required to lift an object with more mass on one side than the other. We expected that greater torque error, poor sensory score, and weaker strength would predict poor performance (i.e., greater total force) on the BMHFT.

## 2. Methods

Thirty-nine typically developing children and adolescents provided assent, and parents provided written informed consent to participate in this study. Participants ranged in age from 5 to 10 years old (*n* = 28) and 15 to 17 years (*n* = 11). The study required each participant to participate in one session lasting approximately 2 h. Participants were right-hand dominant with normal or corrected-to-normal vision, no upper limb injury, and no musculoskeletal or neuromuscular disorders as reported by a parent. The study procedures were approved by the Institutional Review Board of the University of Houston.

### 2.1. Bead Maze Hand Function Test

**Figure 1:**
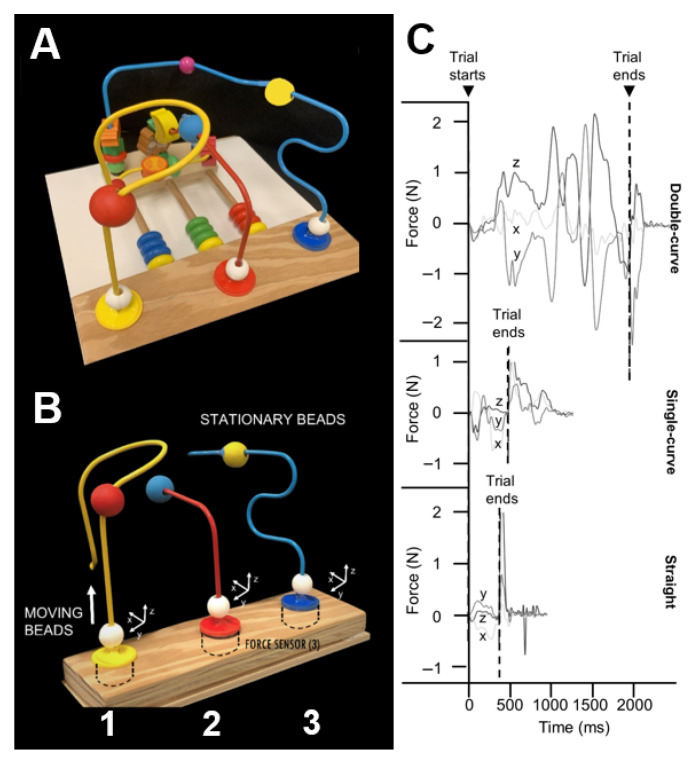
(**A**) Bead Maze Hand function Test apparatus, (**B**) Diagram showing where sensors are placed, moving and stationary beads for each wire shape (straight, single-curve, and double-curve). (**C**) Representative force tracing outputs (horizontal—x, y, and vertical—z) shown in Newtons (N) for each wire shape. Reproduced from Rose et al. 2024 [23].

The BMHFT apparatus (Figure 1A), as previously described [23], includes three triaxial force transducers (Nano 25, ATI, NC; 1000 Hz) mounted on a base beneath a top plate holding straight and curved metal wires. This setup captures forces along all axes (x, y, z; Figure 1B) as participants move beads along the wires. Each wire is rigidly connected to a sensor to allow continuous force recording during movement. Three wire shapes—straight, single-curve, and double-curve—represent increasing levels of difficulty. The moving beads (MVBs) are 18 mm in diameter with a 6.5 mm lumen and have textured surfaces to reduce slippage. Each wire also includes a stationary bead (STB) affixed to the wire at a fixed distance from the sensor. In the present study, we tested only the double curve wire because our previous work noted greater sensitivity of the total force to the precision demands of the complex shape of the double curve wire [23].

Participants sat with their hands resting on their laps, with the BMHFT apparatus placed 10 cm from the table edge. Verbal instructions were to grasp the MVB, lift it, and draw it over the wire until it came in contact with the STB. Once the MVB contacted the STB, participants were asked to release their grasp and return their hand to their lap. After a demonstration and one practice trial, participants used their dominant hand to perform five trials at a self-selected, comfortable speed. No specific instructions were given on grip, contact with the wire, or task execution [23].

### 2.2. Laboratory-Based Dexterous Object Manipulation Task

Digit forces and torques were recorded using a custom inverted T-shaped pediatric device instrumented with two 6-axis force/torque sensors (Nano-25, ATI Industrial Automation, Apex, NC, USA; 1000 Hz), featuring two 10 cm, 320-grit sandpaper grasping surfaces and a 5 cm total width [6]. The base included hidden compartments (left, center, right) for inserting a 200 g external mass, which generated ±122 N·mm frontal-plane torque depending on its position. The total weight including sensors and hidden mass was 421.9 g. Object roll (tilt) was tracked via an electromagnetic sensor (Polhemus FASTRAK; 0.05° resolution, 120 Hz) placed on top. Force data were sampled at 1 kHz via an analog-to-digital converter (PCI-6220 DAQ, National Instruments, Austin, TX, USA).

Participants were instructed to lift the device ~10 cm from the table, pause for ~1 s, then place it back down. They aimed to lift it as vertically as possible to prevent frontal-plane rotation caused by the asymmetrical mass. Before each block of 10 trials, participants were informed that a change was made in the mass location, watched a demonstration, and completed two practice attempts with a centered mass. Between blocks, the external mass was repositioned out of view. Participants had to anticipate—not react to—the torque through successive lifts. Visual feedback from the object’s roll indicated task performance. Over repeated attempts, participants learned to predict and apply compensatory torque before lifting.

### 2.3. Experimental Design

The experiment was conducted during one session in a well-illuminated and quiet room. Participants began with a hand preference test, and their hand span and length were measured [6]. For strength measures, participants were instructed to pinch a standard pinch gauge (accuracy ±1%, B&L Eng, Santa Ana, CA, USA) as hard as possible using a precision pinch grip and key grip. We used the highest score of the two attempts for our analysis. For stereognosis, three familiar objects (key, spoon, pin) and six similar matched objects (e.g., button/coin; paper clip/safety pin; pen/pencil) were placed into the right hand from behind a screen, not visible to the participant. Participants verbally identified or matched each object to a visual display of the same objects, with scores ranging from 0 to 9 [24]. Participants then performed the BMHFT and the dexterous object manipulation task in a counterbalanced order, with ~5 min of rest between the two to avoid fatigue and learning effects [6,8,25].

### 2.4. Data Analysis

#### 2.4.1. Total Force on the BMHF Test

The BMHFT force data were collected via a custom LabVIEW (2016) program (National Instruments, Austin, TX, USA) and processed using a MATLAB (R2025a) script (MathWorks, Natick, MA, USA) with a 30 Hz low-pass, zero-phase lag, and the 5th order Butterworth filter [23]. The DC offset was removed for each trial during data acquisition. Trial start was defined as the moment x, y, or z force exceeded baseline (mean ± 2 SD) for at least 10 ms. Trial end was marked by a stereotypical y-force drop to −0.2 N following MVB-STB contact and before grasp release (Figure 1C). Start and end times were algorithmically identified and visually confirmed. The trial duration was computed as the difference between the trial end time and the trial start time (in sec). The absolute value of the force signals from contact to end was summed to the total force for each trial (the L1 norm). The L1 norm was chosen because it analyzes the influence of individual force components by weighing them equally and is more robust to outliers. Our previous work [23], which systematically investigated within-session learning effects in neurotypical children of similar ages, failed to observe within-session learning effects. The average of 5 trials was used for analysis [23].

#### 2.4.2. Torque Error (TE) on the Dexterous Manipulation Task

The force data were run through a 5th-order low-pass Butterworth filter [6,8,25]. Position data were resampled at the same rate as the force data (MATLAB; MathWorks). Object lift onset was defined as the time at which the vertical position of the grip device crosses and remains above a threshold (mean +2 SD of the baseline) for 200 ms. The torque exerted on the object was computed at the time of lift onset to quantify anticipatory control, which requires not only intact sensory information from the fingers and the ability to execute motor commands, but also the ability to integrate sensory information with motor commands, i.e., sensorimotor integration [3,4]. This is the time before subjects perceive and react to the external torque. The torque error (TE) is the absolute difference between the amount of applied compensatory torque and the target compensatory torque that is required to lift the object vertically. The TE during the last 5 trials of both the right and the left weighted conditions was averaged for each subject.

#### 2.4.3. Statistical Analysis Using Machine Learning

The machine learning analysis used a dataset (*n* = 39) that included features such as TE for right and left weighted conditions, Stereognosis score, Pinch Strength, and Key Strength. In addition to these sensory and motor parameters, we considered age, gender, and hand size (span and length) as potential predictors due to their known influence on an individual’s ability to exert forces on an object [9,23,26,27]. Our prior work demonstrated significant correlations between hand length, hand span, pinch strength, and key strength (see Table 2 in [6]). To account for the issue of multicollinearity, we used the ridge regression (L2 regularization) model to analyze features that influence the total force on the Bead Maze Hand Function Test. A ridge regression (RR) model is a form of multiple linear regression analysis that applies an L2 shrinkage penalty, pulling all predictor coefficients toward zero—though not exactly to zero—with strong predictors shrinking less than weak ones [28,29]. Additionally, it also reduces the effect of collinearity by distributing coefficient weight across correlated predictors. For instance, given *k* correlated variables, each would get identical coefficients equal to 1/*k*th the size that any one variable would get if fit singly [29]. This is due to the regularization parameter λ, added to the standard least square objective function, which controls the overall degree of shrinkage [29]. Thus, RR is an improved least square method that solves multicollinearity among the variables and overfitting in predictive models [30].

Features were first standardized using StandardScaler, and hyperparameters were optimized via GridSearchCV with 5-fold cross-validation (KFold). The dataset was split 75/25 into training and testing sets using stratification [31], and the experimental results were analyzed by RR. Performance of the RR model was evaluated with R^2^, AIC, and BIC. Further, we used Shapley Additive exPlanations (SHAP), a game-theoretic approach to study feature importance for the RR model using all variables ranked by absolute mean values [32,33]. For better interpretability, Spearman correlations between features and predicted scores, with Bonferroni-adjusted *p*-values, assessed bivariate relationships (α < 0.05). The analysis was conducted using Python (version 3.13).

## 3. Results

### 3.1. Ridge Regression Model Performance

The performance of the ridge regression model using all nine features to predict the total force measure on the Bead Maze Hand Function Test was evaluated on training (29 samples) and test (10 samples) datasets. The RR model achieved a good training fit (R^2^ = 0.32) but lower test performance (R^2^ = 0.19). The AIC and BIC of the model were 147.87 and 150.59, respectively.

### 3.2. Feature Importance

**Figure 2:**
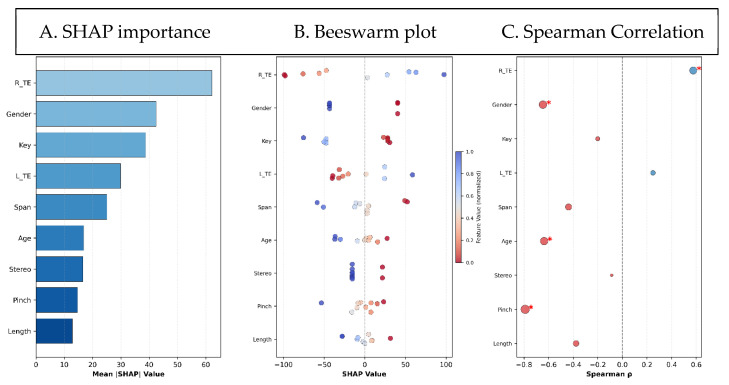
Feature analysis. (**A**) Ridge regression (RR) feature importance ranked by mean absolute SHAP values. (**B**) SHAP beeswarm plot showing feature impacts on RR predictions (color = feature value). (**C**) Correlation between standardized features and ridge regression-predicted total force (for statistically significant features): Negative correlation (red) and Positive correlation (blue). Different sphere sizes represent the magnitude of the correlation. Asterisks indicate corrected *p* < 0.05.

We rank-ordered all nine features based on their absolute SHAP coefficients (Figure 2A) and then examined the correlation of each feature with the total force, applying Bonferroni correction to adjust for multiple comparisons. The beeswarm plot (Figure 2B) shows the distribution of SHAP values and the direction of prediction for each feature. Mainly, a higher TE for the right center of mass condition pushes predictions towards a higher total force on the BMHFT, indicating poor manual performance. Similarly, a male gender pushes prediction toward a higher total force on the BMHFT. In contrast, a lower child’s age and lower pinch strength push prediction toward a higher total force on the BMHFT. Four features exhibited statistically significant Spearman correlations (corrected *p* < 0.05; Figure 2C): TE for the right center of mass condition (absolute mean SHAP = 62.24; Spearman ρ = 0.58; *p* = 0.03), Gender (absolute mean SHAP = 42.47; Spearman ρ = −0.65; *p* = 0.009), Age (absolute mean SHAP = 16.83; Spearman ρ = −0.64; *p* = 0.016), Pinch strength (absolute mean SHAP = 14.69; Spearman ρ = −0.79; *p* = 0.0007).

## 4. Discussion

In this study, we performed a machine learning-based ridge regression analysis to predict the total force output on an activity-based assessment of precision force control, the BMHFT, using measures of strength, stereognosis, and sensorimotor integration (TE), in addition to age, gender, and hand size, in typically developing children and adolescents. We found that the error in torque production while lifting an object with its mass shifted to the right (R_TE) was the best predictor of performance on the BMHFT. Greater R_TE was associated with greater total force on the BMHFT. Of other rank-ordered features, age, gender, and pinch strength showed significant association with the total force on the BMHFT. We discussed these findings in the context of the literature on the development of precision force control and their relevance to how the BMHFT might be used to connect developmental changes in body structures/functions with improvement in fine manual performance.

### 4.1. Linking Lab-Based Assessments to the BMHFT

A key finding of this study is that sensorimotor performance on a fine motor object manipulation task (viz., the right center of mass condition) in a laboratory setting is the best predictor of the force control during an activity-based assessment of hand function. In the grasping literature, fine motor skill is measured by how well individuals can anticipate and adjust their grasp parameters, e.g., accurate scaling of force and torque, based on an object’s properties before lifting it. The anticipatory control reflects the brain’s ability to use sensorimotor memories from previous interactions with the same or similar objects to plan precise, dexterous movements [1,7,8]. In the object manipulation task, participants were required to scale their torque based on the presumed center of mass before lifting the object, with a goal to minimize tilt. Torque error was calculated as the deviation from the ideal torque needed to lift the object without tilting. Smaller or no deviation from the target torque (i.e., torque error or TE ~0 Nˑmm) indicated accurate anticipatory control, while a larger TE results in a greater reliance on sensory feedback to correct the movement, a phenomenon known as sensorimotor integration [1,8,25]. In younger children, the ability to apply accurate torque may also depend on the task demands, such as whether the object’s center of mass is shifted to the right or left [6]. Children between 8 and 10 years of age can modulate their grasp parameters when performing the object manipulation task in the left-shifted mass condition, but failed to do so in the right-shifted mass condition [6]. Smaller hands and lower strength might make it mechanically difficult to transmit force with the index finger when using the thumb as a pivot. The BMHFT, on the other hand, requires a combination of anticipatory and reactive strategies for the control of grasp parameters. As the child grasps and moves the bead along the wire, they encounter several curves that require anticipatory tilting and rotating of the bead with their digits to ensure smooth movement around it. Anticipatory tilting of the bead is crucial; without it, the bead rubs against the curves in the wire, increasing force on the wire, which can limit its progression along the path. Our findings suggest that participants who were better able to control their digit forces in an anticipatory manner (less TE) were better able to smoothly navigate the bead along the curves, imparting less force onto the wire.

### 4.2. Linking Demographics and Clinical Measures to the BMHFT

Consistent with our earlier work [6,23], the total force on the BMHFT decreased with age. Lower total force during the double curve wire in older children might be due to better ability to orient the bead relative to the wire, possibly due to improved steadiness of contractions of hand/forearm/arm muscles with age [34]. Consistent with normative data for the 9-Hole Peg Test and the MABC-2 dexterity subtests, females demonstrated superior performance on the BMHFT compared to males [27,35]. Similar gender-related patterns have been reported in studies of novel manual-skill acquisition; for example, girls aged 4–11 years showed better accuracy than boys in a stylus-based tracing task [36]. These converging findings suggest that future large-scale normative testing of the BMHFT should determine whether gender-specific reference values are warranted.

Impairments in grip/pinch strength, moving 2-point discrimination, stereognosis, and spasticity have been shown to predict scores on clinical tests such as the Assisting Hand Assessment, Jebsen Taylor Hand function test, and Pegboard test [24,37]. In another model, two-point discrimination, spasticity, and pinch strength are best related to the ability of children with unilateral spastic cerebral palsy to adapt and scale their grip force during prehensile tasks [9]. The relationships between sensorimotor parameters and dexterity vary according to the specific parameters measured and the tasks involved in each assessment. Collectively, these studies highlight the value to clinicians in understanding which sensorimotor measures best relate to the performance of a given dexterity test. For typically developing children and adolescents, we found maximum pinch grip strength as one of the predictors of the total force on the BMHFT. Greater pinch strength was significantly associated with better performance on the BMHFT. This result was somewhat surprising given that the BMHFT requires very little strength. The bead is small, lightweight, and slides freely over the metal wire; very low forces are required to manipulate the bead. We speculate that greater maximum pinch strength, predicting lower total force on the BMHFT, can be attributed to the development of independent force control for precision pinch [38], the primary grip type chosen to manipulate the bead. Producing a maximum pinch force requires the ability to isolate and direct fingertip forces, a capability that improves with age [39]. Maintaining isometric precision pinch force across varying percentages of maximum voluntary contraction requires the development of neuronal connections and pathways [40] to isolate [41] and scale the forces of the specific muscles involved in executing an isometric precision pinch [38,39,42].

Stereognosis was not a significant contributor to performance on the BMHFT for children and adolescents. In typically developing children, it may not be possible to test whether stereognosis plays a role in performance. A larger sample, younger ages, and/or an alternative measure of tactile sensibility would be required. Given the importance of online sensory feedback during dexterous manipulation tasks [8,10], future work will include more sensitive or graded tactile sensibility testing to study subtle developmental changes in sensibility and the resulting impact on the BMHFT measures.

The study’s findings also extend beyond the immediate research context, highlighting broader applications in clinical settings. By suggesting the importance of force control as a sensitive measure for assessing manual skill, our study suggests potential utility in detecting changes in motor skill post-surgery or therapeutic intervention. Additionally, exploring other predictors or moderators of BMHFT performance, such as cognitive factors or tactile sensibility, could provide a more comprehensive understanding of the factors influencing performance. Further validation studies of the BMHFT in clinical populations could also be explored to address this objective, as well as investigating additional psychometric properties of the BMHFT, such as test–retest reliability and responsiveness to change. By refining our understanding of fine motor function during development, we can optimize rehabilitation strategies and ultimately improve outcomes for children with impaired fine motor skills.

### 4.3. Limitations

One of the limitations of this study is the lack of participants in preadolescence, ages 12–14 years. Thus far, the primary focus of the BMHFT has been quantifying performance in the age ranges of 4–10 years, when force control for manipulating objects is known to develop. Additionally, based on the parallel development of dexterity scores and neuroanatomical development [42], we expect that additional participants aged 12–14 years would not significantly change the relationship between total force and age. Despite the smaller sample size in pre-adolescence, the findings from this work support larger psychometric studies in clinical populations. The present study focused on the double-curve wire on the BMHFT because our previous work noted greater sensitivity of the total force to the precision demands of the complex shape of the double-curve wire [23]. A broader generalizability across wire types is an important future direction, especially for studies involving clinical or developmental populations where strategy shifts may be more pronounced.

### 4.4. Conclusions

Our findings highlight the importance of a force control measure in clinical assessment of hand functions for daily tasks that require precision. Specifically, we observed how sensorimotor capabilities of anticipatory control and maximum precision pinch strength can predict performance on the BMHFT, an activity-based assessment of hand function. The preliminary evidence shows correlations of TE and pinch strength with BMHFT total force, suggesting that function-oriented assessments centered on force control may have potential value. A better understanding of how the development of sensorimotor capabilities influences treatment outcomes can aid in the refinement of rehabilitation protocols and treatments [5,43,44,45,46,47,48]. For clinicians, this could mean a more complete evaluation of a child’s hand function and better-informed intervention planning. The findings suggest further development of sensitive, force-based measures to improve outcomes for children who struggle with fine motor tasks.

## Data Availability

The data presented in this study are available upon reasonable request.
